# Eigenvalue calibration method for 3 × 3 Mueller polarimeters

**DOI:** 10.1364/OL.44.002362

**Published:** 2019-04-30

**Authors:** Ji Qi, Daniel S. Elson, Danail Stoyanov

**Affiliations:** 1 Wellcome/EPSRC Centre for Interventional and Surgical Sciences, University College London, London W1W 7TS, UK; 2 Nanophotonics Research Centre, Shenzhen University, Shenzhen 518060, China; 3 Centre for Medical Image Computing, University College London, London W1W 7TS, UK; 4 Department of Computer Science, University College London, London W1W 7TS, UK; 5 Hamlyn Centre for Robotic Surgery, Imperial College London, London SW7 2AZ, UK; 6 Department of Surgery and Cancer, Imperial College London, London SW7 2AZ, UK; 7 e-mail: daniel.elson@imperial.ac.uk; 8 e-mail: danail.stoyanov@ucl.ac.uk

## Abstract

3×3 Mueller polarimetry has shown potential for tissue characterization applications, however, calibration has not been fully addressed. We demonstrate a 3×3 Mueller polarimeter eigenvalue calibration method, inspired by those for full Mueller polarimeters. We also investigate the optimal combination of calibration measurements. Our method does not rely on modeling the polarization state generator, polarization state analyzer, or precise knowledge of calibration sample properties or orientations. It is therefore easy to implement, and the experimental results of a linear polarizer test sample, as well as a biological specimen, are presented.

3×3 Mueller polarimetry measures the top left 3×3 sub‐matrix of a 4×4 Mueller matrix, conveying a substantial proportion of the sample polarization properties [1–3]. Partial Mueller matrices can be determined without using phase retarders, simplifying the system and measurement procedures by only using linear polarizers. 3×3 Mueller polarimetry has been demonstrated as feasible in several potential applications, including endoscopy [4–9].

The polarization state generator and analyzer (PSG/PSA) of a 3×3 Mueller polarimeter only involve linear polarizers (LPs). The 4×4 Mueller matrix of a general LP is given by

$$\begin{array}{l} \;M_{\mathrm{LP}}=\left[\begin{array}{cc} \mathrm{Rot}(\theta ) & 0\\ 0 & 1 \end{array}\right]\left[\begin{array}{cccc} q+r & q-r & 0 & 0\\ q-r & q+r & 0 & 0\\ 0 & 0 & 1 & 0\\ 0 & 0 & 0 & 1 \end{array}\right]\left[\begin{array}{cc} \mathrm{Rot}(-\theta ) & 0\\ 0 & 1 \end{array}\right]\\ \mathrm{Rot}(\theta )=\left[\begin{array}{ccc} 1 & 0 & 0\\ 0 & \cos\;\theta & \sin\;\theta \\ 0 & -\sin\;\theta & \cos\;\theta \end{array}\right], \end{array}$$ (1)

where θ is the orientation angle, and q and r are the maximum and minimum attenuations along two principal axes of the LP. In practice, θ, q, and r of the LPs used in the PSG and PSA of 3×3 Mueller polarimeters may deviate from their nominal values. The light source (considered as a part of the PSG) may also not be perfectly unpolarized. Therefore, it is important to develop a calibration method to obtain the actual PSG and PSA instrumental matrices.

A traditional calibration method uses an additional LP with the orientation precisely controlled, an unpolarized light source, and a detector, thereby calibrating the orientation of the LPs within the PSG and PSA, based on the established null intensity calibration method (NICM) [4,5,10]. This emphasizes the calibration of θ for the LPs, rather than q and r. Ignoring the calibration of q is problematic, especially for PSGs/PSAs with multiple LPs such as division-of-focal-plane PSAs or division-of-amplitude PSAs [4,6] that usually have different q values. Ignoring the calibration of r might lead to errors, e.g., in multispectral polarimetry, where r may not be 0 for all wavelengths. Obtaining an additional unpolarized light source and precisely controlling the orientation of the additional LP also requires extra time and effort. Another calibration method [6] uses nine gain coefficients obtained by fitting data for an additional rotating LP to correct radiometric measurements. This method assumes that the transformation matrix between the actual and the nominal PSG/PSA matrix is diagonal, which is normally not the case.

Here we demonstrate a calibration method for 3×3 Mueller polarimeters inspired by that for complete Mueller polarimeters [11]. This method does not require to (1) model the PSG/PSA, so θ, q, and r of the PSG/PSA LPs, and unpolarized light sources are calibrated altogether; or (2) precisely know the properties and orientations of the calibration samples (CSs). This method is easy, quick, and convenient to implement.

**Eigenvalues of calibration measurements.** In complete Mueller polarimetry, the Mueller matrix is solved from [2]:

$$P=M_{\mathrm{PSA}}M_{\left(4\times 4\right)}M_{\mathrm{PSG}},$$ (2)

where $M_{\mathrm{PSA}}$, $M_{\mathrm{PSG}}$, $M_{\left(4\times 4\right)}$, and P are the PSG/PSA instrumental matrices, complete Mueller matrix of a sample, and radiometric intensity matrix, with each element corresponding to each individual PSG/PSA state. In 3×3 Mueller polarimetry, because only LPs are used, the fourth columns of $M_{\mathrm{PSA}}$/$M_{\mathrm{PSG}}$ are zero. Equation (2) can be rewritten in partitioned form as

$$\begin{array}{l} P_{\left(a\times g\right)}=\left[\begin{array}{cc} A_{\left(a\times 3\right)} & 0_{\left(a\times 1\right)} \end{array}\right]\left[\begin{array}{cc} M_{\left(3\times 3\right)} & X_{a(3\times 1)}\\ X_{b(1\times 3)} & X_{c(1\times 1)} \end{array}\right]\left[\begin{array}{c} G_{\left(3\times g\right)}\\ 0_{\left(1\times g\right)} \end{array}\right]\\ \;=A_{\left(a\times 3\right)}M_{\left(3\times 3\right)}G_{\left(3\times g\right)}. \end{array}$$ (3)

A and G are the reduced PSA and PSG instrument matrices for a 3×3 Mueller polarimeter, $M_{\left(3\times 3\right)}$ is the 3×3 Mueller sample matrix. Symbols and numbers inside brackets stand for the matrix or matrix partition. The calibration can be considered as a process to obtain A and G from the radiometric measurements (P) of CSs whose $M_{\left(3\times 3\right)}$ are not precisely known. Equation (3) has the same form as Eq. (2) and lays the foundation to extend the eigenvalue calibration method of complete Mueller polarimeters to 3×3 Mueller polarimeters.

The null response of the polarimetric system can be acquired by measurements of air, represented by

$$P_{\text{air}}=\mathrm{AG}.$$ (4)

A calibration measurement $P_{i}$ is characterized by

$$P_{i}={\mathrm{AM}}_{i(3\times 3)}G,$$ (5)

in which $M_{i(3\times 3)}$ is the 3×3 Mueller matrix of the CS. An intermediate matrix $D_{i}$ can then be constructed:

$$D_{i}=P_{{\text{air}}^{-1}}P_{i}=G^{-1}M_{i(3\times 3)}G.$$ (6)

According to Eq. (6), $D_{i}$ and $M_{i(3\times 3)}$ are similar matrices and have the same eigenvalues.

**Characteristics of CSs.** Inspired by Ref. [11], we explored using the polarization components that follow a dichroic retarder (DR) model as the CS, which has form

$$\begin{array}{l} \left[\begin{array}{cc} \mathrm{Rot}(\theta ) & 0\\ 0 & 1 \end{array}\right]\left[\begin{array}{cccc} q+r & q-r & 0 & 0\\ q-r & q+r & 0 & 0\\ 0 & 0 & 2\sqrt{qr}\;\cos\;\Delta & 2\sqrt{qr}\;\sin\;\Delta \\ 0 & 0 & -2\sqrt{qr}\;\sin\;\Delta & 2\sqrt{qr}\;\cos\;\Delta \end{array}\right]\\ \;\times \left[\begin{array}{cc} \mathrm{Rot}(-\theta ) & 0\\ 0 & 1 \end{array}\right]. \end{array}$$ (7)

θ, Δ, q, and r are the orientation, retardance, and attenuation along the two principal axes of the DR. When q>r, and Δ=0, the DR reduces to a LP. When q is similar to r, the DR reduces to a linear retarder with retardance Δ.

The eigenvalues of $M_{\left(3\times 3\right)}$ for a CS should also contain the necessary information such that $M_{\left(3\times 3\right)}$ can be partially constructed, another important premise to extend 4×4 Mueller polarimeter eigenvalue calibration to the 3×3 case. It is easy to derive Eq. (8) from Eq. (7) based on partition matrix multiplication to obtain the 3×3 Mueller matrix $M_{i(3\times 3)}$ of a DR with orientation θ given by

$$M_{i(3\times 3)}=\mathrm{Rot}(\theta )\left[\begin{array}{ccc} q+r & q-r & 0\\ q-r & q+r & 0\\ 0 & 0 & 2\sqrt{qr}\;\cos\;\Delta \end{array}\right]\mathrm{Rot}(-\theta ).$$ (8)

Thus, the three eigenvalues of $M_{i(3\times 3)}$ for a DR are independent of θ and can be obtained analytically by

$$\lambda_{1}=2q,\lambda_{2}=2r,\lambda_{3}=2\sqrt{qr}\;\cos\;\Delta .$$ (9)

Thus, it is possible to partially reconstruct $M_{i(3\times 3)}$ (only with θ undetermined) of the CS from the eigenvalues of the intermediate matrix $D_{i}$.

**Calibration of polarimeters.** Equation (6) is rewritten as:

$${\mathrm{GD}}_{i}-M_{i(3\times 3)}G=0,$$ (10)

and can be solved by matrix vectorization and diagonalization [11]. After G’s columns are stacked into a column vector (vec(G)), Eq. (10) is transformed to Kronecker product (denoted by ⊗) form [12]:

$$\begin{array}{l} H_{i}\;\text{vec}(G)=0\\ \;H_{i}=D_{i(g\times g)}⊗I_{\left(3\times 3\right)}-I_{\left(g\times g\right)}⊗M_{i(3\times 3)}. \end{array}$$ (11)

I is an identity matrix. To ensure that $H_{i}$ is diagonalizable, Eq. (11) is multiplied on both sides by $H_{i}^{T}$ to construct a Hermitian matrix:

$$H_{i}^{T}H_{i}\;\text{vec}(G)=0.$$ (12)

Obviously, vec(G) exists in the eigenspace of $H_{i}^{T}H_{i}$ which corresponds to the null eigenvalue. vec(G) can be uniquely determined by choosing a suitable CS/measurement so that the constructed matrix K in Eq. (13) only has one null eigenvalue:

$$K\;\text{vec}(G)=0,\;\mathrm{where}\;K=\underset{i=1,2,\ldots ,n}{\sum }H_{i}^{T}H_{i}.$$ (13)

The subscript i is the sequence number of each CS/measurement. It is noted that $M_{i(3\times 3)}$, along with $H_{i}$ and K, is partially determined with the orientation $\theta_{i}$ of each unknown CS. $\theta_{i}$ can be determined through optimization by finding those corresponding to the minimal ratio between the smallest and second smallest eigenvalues of K denoted by μ1 and μ2, respectively [11]:

$$\theta_{i=1,2,\ldots ,n}=\underset{\theta_{i=1,2,\ldots ,n}}{\mathrm{arg min}}\sqrt{\frac{\mu 1}{\mu 2}}.$$ (14)

K and G, can then be fully determined from Eq. (13). With G obtained, A can be calculated from Eq. (4).

**The optimal combination of calibration measurements.** It is crucial to find a combination of CSs/measurements, so that G can be uniquely and accurately determined from Eq. (13). It is therefore necessary to balance the nonzero eigenvalues of K, i.e., maximizing the ratio (referred to as *SSLE-R*) between the second smallest and largest eigenvalues [11]. We restricted the CS to readily available LPs and imperfect quarter-wave plates (QWPs), whose retardance may not be 90°. Two PSG/PSA congifurations for a 3×3 Mueller polarimeter were studied. The first PSG generates 0°, 60°, and 120° linearly polarized light with 0°, 60°, and 120° LPs as analyzers, referred to as a three-state PSG/PSA here. This configuration minimizes the number of radiometric acquisitions to reconstruct [2]. The second PSG generates 0°, 45°, 90°, and 135° linearly polarized light with 0°, 45°, 90°, and 135° linear analyzers, referred to as four-state PSG/PSA [13].

It is found that at least one LP measurement is required to guarantee that G can be uniquely determined. Nine possible Confs. of the combination of CS have been explored, as shown in Table 1. The LP in the first calibration measurement is always oriented at 0° by definition. The optimal orientations of CSs for Confs. (1)–(9) were first investigated individually.

**Table 1.** Optimal Orientations (in Degree) of Calibration Samples

Configuration	Number of CS	First Sample		Second Sample		Third Sample		Fourth Sample		Maximum *SSLE-R*
	Involved	Type	$\theta_{1}({}^{\circ})$	Type	$\theta_{2}({}^{\circ})$	Type	$\theta_{3}({}^{\circ})$	Type	$\theta_{4}({}^{\circ})$	
1	2	LP (ER 100:1)	0	LP(ER 100:1)	62	N.A.		N.A.		5.9e−05
2	2	LP (ER 100:1)	0	QWP	28	N.A.		N.A.		5.0e−05
3	3	LP	0	LP	45	LP	135	N.A.		0.0875
4	3	LP	0	LP	90	QWP	117	N.A.		0.0588
5	3	LP	0	QWP	19	QWP	162	N.A.		0.1198
6	4	LP	0	LP	45	LP	90	LP	135	0.2474
7	4	LP	0	LP	90	LP	135	QWP	135	0.1573
8	4	LP	0	LP	145	QWP	8	QWP	140	0.1268
9	4	LP	0	QWP	22	QWP	55	QWP	77	0.1677

*SSLE-R* of K is considered as a function of the orientation angles of the CS in the process of finding the optimal combination of the CSs. The optimal combination can then be obtained by finding the argument of the *SSLE-R* function corresponding to the maximum *SSLE-R* value.

The optimal orientations of CSs and maximum *SSLE-R* value under each configuration is provided in Table 1. For Confs. 1 and 2, K has more than one null eigenvalue when the LP has infinitely large extinction ratio (ER). G would be solvable when LP has finite ER. Here we assumed the LP has ER 100:1 for Confs. 1 and 2. In general, the maximum *SSLE-R* value under Confs. 1 and 2 is three orders of magnitude lower than the other configurations. Therefore, any combination of CSs under Confs. 1 and 2 is extremely sensitive to noise and is not the optimal Confs. For Confs. 3–5, which all entail three measurements of CS, Conf. 5 has the higher maximum *SSLE-R* value at 0.1198. As shown in Fig. 1(a), one of the maximum *SSLE-R* value for Conf. 5 is achieved by using a 19° and 162° QWP, together with 0° LP. Among the four CS measurement Confs. 6–9, Conf. 6 has the maximum *SSLE-R* value. The maximum *SSLE-R* value corresponding to 0°, 45°, 90°, and 135° LP is as large as 0.2474 [Fig. 1(b)]. This combination is the optimal among all the configurations explored in this Letter. It is also found that the three-state (0°, 60°, and 120°) and four-state PSG/PSA (0°, 45°, 90°, and 135°) results are the same.

**Fig. 1.**
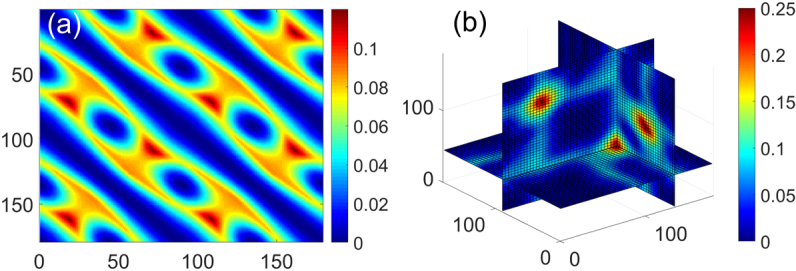
*SSLE-R* values correspond to all the possible orientations of the CSs in (a) Conf. 5 and (b) Conf. 6. The color represents the *SSLE-R* value. Note that the first sample is a 0° LP. The axes in (a) represent the orientation of the second sample (QWP) and third sample (QWP) under Conf. 5. The three axes in (b) are the orientation of the second (LP), third (LP), and fourth samples (LP).

We took experimental errors into account by adding synthetic Gaussian noise to Eqs. (4) and (5) with a controlled amplitude (0.5% was adopted with reference to [11]), as shown below:

$$P_{i\_\text{noise}}=P_{i}+0.5\%\|Pi\|\text{randn},$$ (15)

randn represents Gaussian distributed random numbers with zero mean and standard deviation 1, and the double bar refers to the matrix Fronius norm. Calibration errors for PSG/PSA are defined as

$$\varepsilon_{G}=\|G_{\mathrm{cal}}-G\|/N_{G},\varepsilon_{A}=\|A_{\mathrm{cal}}-A\|/N_{A}.$$ (16)

$N_{G}$ and $N_{A}$ denote the number of elements in G and A. The subscript cal denotes the instrument matrices after the ECM. The calibration errors for each combination of CSs were obtained by simulating the calibration process 100 times. A four-state PSG/PSA was used in this simulation.

The general trend is that the calibration error decreases with the rise of the *SSLE-R* value as expected. The mean calibration errors for Confs. 1 and 2 are about 0.097 and 0.870, respectively, several orders of magnitude higher than the others and should not be used for calibration. The errors for Confs. 3–9 are shown in the boxplots in Fig. 2. The errors involving four CSs (Confs. 6–9) are generally smaller than for three CSs (Confs. 3–5). The minimal calibration error among all Confs. is achieved by adopting 0°, 45°, 90°, and 135° LPs as CSs under Conf. 6. Among all the configurations involving three CSs, the combination specified under Conf. 5 demonstrated the smallest calibration error. Conf. 5 may occasionally have more advantages over Conf. 6, since it needs fewer measurements and less computation, because the optimization in Eq. (14) involves two variables, while Conf. 6 involves three.

**Fig. 2.**
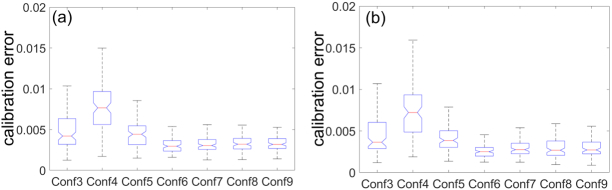
Errors of (a) PSG and (b) PSA instrumental matrices obtained by simulating the calibration process 100 times with a noise amplitude (0.5%) for Confs. 3–9. The errors for Confs. 1 and 2 are several orders of magnitude higher and are not shown.

**Experiment with an LP and a tissue slide.** A 3×3 Mueller polarimeter with the four-state PSG/PSA in transmission geometry was set up to validate the method. The light source was a collimated LED (MCWHLP1, 2350 mW white light, Thorlabs) with a bandpass filter (546DF10, central wavelength 546 nm and bandwidth 10 nm, Omega Optical). The PSG of this setup was an LP (TECHSPEC, Edmunds Optics) driven by a motorized rotation mount (PRM1/MZ8, Thorlabs). The PSA consisted of four LPs contained in automated filter wheels (FW103H/M, Thorlabs). The orientations of the four LPs in the PSA were aligned with the PSG by using the method in Ref. [5], referred to as the NICM. The switching times of the PSG/PSA were 4000 and 55 ms, respectively and the cooled CCD camera (Retiga Exi, QImaging) exposure time was 4 ms with 500 ms waiting time to ensure the PSG/PSA had switched. One image was taken for each PSG/PSA state. The total acquisition time was 26 s for a 3×3 Mueller polarimetric image.

A high-contrast glass LP (Edmund Optics, nominal ER 10000:1 at 500–600 nm) was initially used as a test sample. The combination of CSs under Conf. 6 was used to calibrate based on the ECM. The results were then applied to data of the high-contrast LP sample rotated from 0° to 350° in 10° steps. Each subplot in Fig. 3(a) was obtained by using the calibrated 3×3 Mueller matrices of the rotating LP subtracted from the actual ones. It is obvious that the residual errors of the rotating LP sample with the polarimeter calibrated using the ECM are smaller than those using the NICM. The residual average elemental error was defined by

$$\varepsilon_{\mathrm{ECM}}=\|M_{\mathrm{ECM}}-M\|/9,\;\varepsilon_{\mathrm{NICM}}=\|M_{\mathrm{NICM}}-M\|/9.$$ (17)

As demonstrated in Fig. 3(b), the residual errors of the rotating LP sample with the polarimeter calibrated using the ECM are about one-third of those using the NICM.

**Fig. 3.**
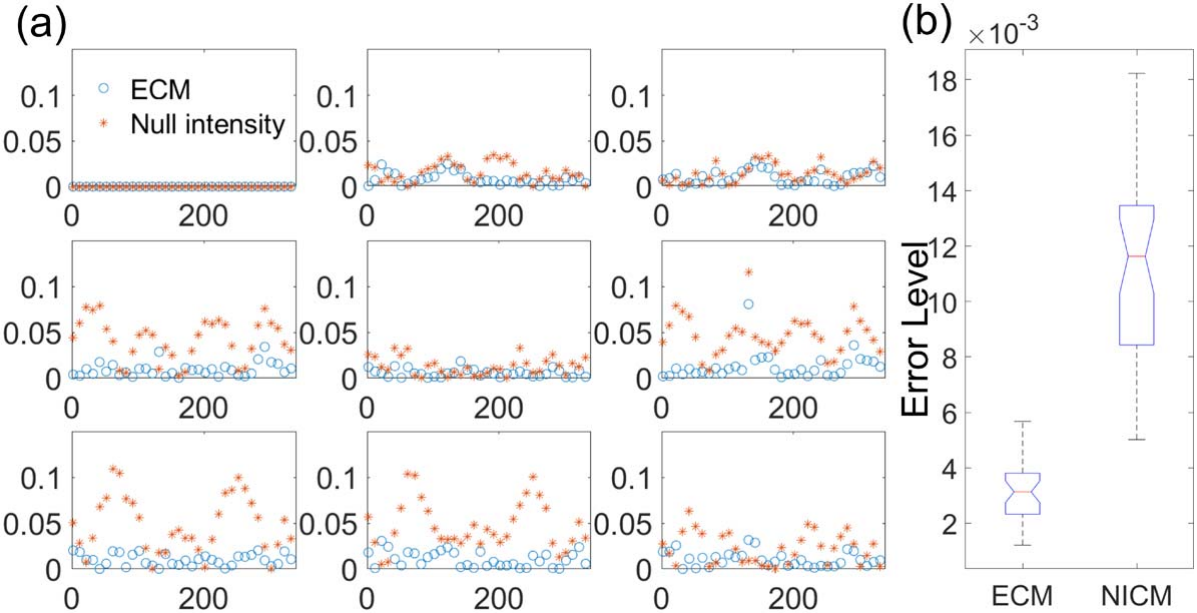
(a) Residual errors of the rotating LP sample with the polarimeter calibrated using the ECM in blue and those with NICM in red. Each subplot corresponds to an element of 3×3 Mueller matrices. The horizontal and vertical axes refer to the angle of the LP sample and error. (b) Boxplot of residual errors.

We then adapted a polarization microscopy objective lens (×10, UPLFLN-P Olympus) to the calibrated 3×3 Mueller polarimeter [Fig. 4(a)]. Porcine esophagogastric junction was obtained for imaging. The tissue appeared slightly yellowish and hardened associated with frequent acid reflux (non-malignant) and was prepared into 6 μm thick slides. The epithelium area was imaged and 3×3 Mueller images with the ECM and NICM are displayed in Figs. 4(b) and 4(c). The M32 image with the NICM implemented demonstrated a small deviation from 0. In comparison, the one with the ECM is close to 0. The tissue anisotropy image was reconstructed from Figs. 4(b) and 4(c) based on the “*A*” parameter in a Mueller matrix transformation method [3]. The epithelium demonstrates anisotropy [Fig. 4(d)], which might be caused by slight fibrosis. It is noted that the reconstructed anisotropy image with the ECM and the NICM had different values, as shown in the histogram of the subtraction between the anisotropy image with the ECM and NICM in Fig. 4(e). The ECM could contribute to the acquisition of more accurate polarimetric data and quantitative information about tissue anisotropy, which has been used to characterize tissue microstructure\ [14].

**Fig. 4.**
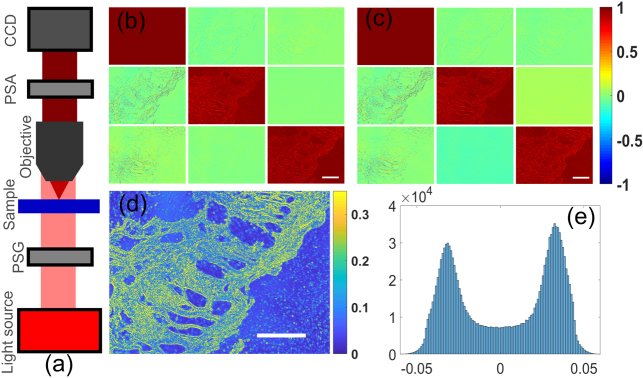
(a) Setup of the 3×3 Mueller polarimetric microscope; (b), (c) the 3×3 Mueller images (1392×1040 pixels) with the ECM and NICM, respectively; the scale bar represents 0.15 mm; (d) reconstructed tissue anisotropy image; the scale bar represents 0.15 mm; (e) histogram of the subtraction between the anisotropy image with the polarimeter calibrated with the ECM and NICM.

In summary, a calibration method for 3×3 Mueller polarimeters has been developed. One of the optimal combinations of calibration measurements is to use LPs at 0°, 45°, 90°, and 135°. There is no need to model the PSG, PSA, or the light source, or to precisely know the properties and orientations of the CSs. Therefore, the method is easy, quick, and convenient to implement.
